# A Rapid Procedure for Identification and Antifungal Susceptibility Testing of Yeasts From Positive Blood Cultures

**DOI:** 10.3389/fmicb.2018.02400

**Published:** 2018-10-15

**Authors:** Alessandra Vecchione, Walter Florio, Francesco Celandroni, Simona Barnini, Antonella Lupetti, Emilia Ghelardi

**Affiliations:** ^1^Dipartimento di Ricerca Traslazionale e delle Nuove Tecnologie in Medicina e Chirurgia, Università di Pisa, Pisa, Italy; ^2^Azienda Ospedaliero-Universitaria Pisana, Pisa, Italy

**Keywords:** yeast, *Candida* species, antifungal susceptibility testing, MALDI-TOF, Sensititre YeastOne Y010, blood culture

## Abstract

The development of rapid diagnostic assays for the identification and analysis of antimicrobial resistance of fungal pathogens causing invasive mycoses is of utmost importance to reduce morbidity and mortality. We evaluated the performance of a novel rapid procedure directly applied to monomicrobial blood cultures from patients with bloodstream infection caused by yeast species, including nine *Candida* and three non-*Candida* species. For the rapid procedure herein developed, samples of positive blood cultures were transferred into serum separator tubes and treated with sodium dodecyl sulfate; the yeast layer was recovered and directly used for microbial identification by MALDI-TOF mass spectrometry and antifungal susceptibility testing (AFST) by the Sensititre YeastOne Y010 panel. The results were compared with those obtained by the same methods applied to colonies isolated on solid media. Using a score value of 1.700 as cut-off for valid identification, the rapid procedure identified 66 of 124 (53.2%) isolates, all of which concordantly with the reference method. However, adopting a cut-off ≥1.300 and ≥4 consecutive repetitions of the same species in the list of matches would extend concordant identification to 107/124 (86.3%) samples. Importantly, AFST revealed essential agreement between the two methods for all the isolate/antifungal drug combinations tested, including misidentified and not identified isolates. Therefore, the procedure herein developed represents a valid alternative for AFST of yeasts from positive blood cultures, yielding accurate and reliable results at least 24 h earlier than with the routine procedure, thus allowing clinicians to promptly streamline antifungal therapy.

## Introduction

Bloodstream infections (BSIs) are among the main causes of morbidity and mortality in hospitalized patients (Buehler et al., 2016). In this context, invasive *Candida* infections are associated with frequently unfavorable outcomes (Haltmeier et al., 2015) and mortality rates higher than 60% in critically ill patients (Kett et al., 2011; Guo et al., 2013; Barchiesi et al., 2014, 2017), especially when they are not timely and appropriately treated (Garey et al., 2006). In general, yeast identification is clinically relevant since different *Candida* species may differ in virulence and drug resistance. For example, the antimicrobial susceptibility pattern of *Candida parapsilosis* and *Candida glabrata* can be quite different, as *C. glabrata* is more frequently resistant to azoles and *C. parapsilosis* to echinocandins (Silva et al., 2012). Moreover, invasive trichosporonosis is characterized by resistance to amphotericin and echinocandins, and poor prognosis (Miceli et al., 2011). Therefore, the ability to rapidly identify these yeasts might be useful to promptly streamline empirical antimicrobial therapy. However, the recent emergence of multidrug resistant (MDR) fungal pathogens (Lamoth et al., 2018), with varying susceptibility profiles to azole drugs, amphotericin B and echinocandins, poses a pressing need for rapid antifungal susceptibility testing (AFST).

Blood culture (BC) is the gold standard for the identification (ID) and antimicrobial susceptibility testing (AST) of the infectious agent(s) causing BSI. The currently applied procedure requires that positive BCs are sub-cultured on solid media to perform ID and AST by automated systems, thus requiring at least 48–72 h from BC positivity to results report. Aiming at shortening the turnaround time for the diagnosis of systemic fungal infections, the present study was undertaken to evaluate the performance of a rapid procedure for the direct identification and AFST of yeasts from positive BCs.

## Methods

### Study Population

Blood cultures resulting positive for yeasts were collected at the Microbiology Unit of the Pisa University Hospital from January 2016 to June 2017. For each patient, only the first positive BC that appeared monomicrobial for yeasts at the Gram-staining was included in this study. In total, 124 BCs were analyzed. The study was notified to the local ethical committee, Comitato Etico di Area Vasta Nord Ovest, University of Pisa, and conducted in full accordance with the principles of the Declaration of Helsinki. Samples were taken as part of the standard patient care, and anonymized by the clinical personnel. Research personnel received and used these samples anonymously. For this type of study, no written informed consent was necessary.

### Yeast Identification

For the rapid procedure, 4 mL of positive BC were transferred into a serum separator tube (SST) (BD Vacutainer System) and treated with 0.5% sodium dodecyl sulfate, to obtain lysis of blood cells. The suspension was briefly vortexed and centrifuged (2,000 × *g*, 2 min). The supernatant was removed, yeast cells were collected from the surface of the silicon layer of the SST and suspended in sterile water. Yeasts were washed twice with sterile water to remove SDS traces, spotted onto the MALDI-TOF target plate, and exposed to an *in situ* protein extraction by addition of 1 μL ethanol, 1 μL 70% formic acid, and 1 μL acetonitrile. After air-drying, the sample was overlaid with 1 μL α–cyano-4-hydroxycinnamic acid (HCCA) solution (matrix) and MALDI-TOF analysis was performed using a Microflex LT mass spectrometer (Bruker Daltonics, Bremen, Germany). Spectra were analyzed using a Bruker Biotyper 3.1 software and library (Bruker Daltonics). Yeast identification was considered reliable with both score values ≥1.7 and ≥4 consecutive repetitions of the same species in the list of matches. In parallel, a routine procedure using yeasts isolated on selective and chromogenic agar media followed by MALDI-TOF MS was also carried out as the reference method. In the overall, nine *Candida* species and *Cryptococcus neoformans*, *Rhodotorula mucilaginosa*, and *Magnusiomyces capitatus* were identified.

### Antifungal Susceptibility Testing

For the rapid procedure, an aliquot of the microbial suspension used for yeast identification was diluted in sterile water to a final density of 0.5 McFarland (2–5 × 10^6^ CFU/mL) and inoculated (20 μl) into the Sensititre YeastOne broth (Remel Inc., Thermo Fisher Scientific, United Kingdom) following the CLSI M27-S4 method (Clinical and Laboratory Standards Institute, 2012). A 100 μl aliquot was dispensed in each well of the Sensititre YeastOne Y010 panel (TREK Diagnostic System, Thermo Fisher Scientific, United Kingdom). The panel was incubated at 35 ± 2°C for 24 and/or 48 h and microbial growth was visually inspected as a change in color from blue (negative) to red (positive) for minimal inhibitory concentration (MIC) determination. The results of the rapid AFST were compared with those obtained by the same assay on isolated colonies grown on selective and/or chromogenic media.

## Results

Using the rapid procedure, 66 of the 124 (53.2%) yeast strains were identified with score values ≥1.700. Even though not generally considered valid for the low scores, we observed that 41 (33.1%) strains were correctly identified with scores between 1.300 and 1.699 and ≥4 consecutive repetitions of the same species in the list of matches. In addition, 2 (1.6 %) strains were correctly identified with scores <1.300 (one *Candida albicans* and one *Candida inconspicua* strain; **Table 1**). Eleven *C. albicans* and two *C. parapsilosis* were not identified and two yeasts were misidentified: one *C. glabrata* was misidentified as *Candida krusei* with score value 1.200 and two consecutive identical species ID in the list of matches, and one *Candida orthopsilosis* was misidentified as *Candida lusitaniae* with score value 1.130 and two consecutive identical species ID.

**Table 1:** Rapid identification of yeasts directly from positive blood cultures by MALDI-TOF MS^∗^.

		Correct identification with score				No identification	Misidentification
Species	tot	<1.300	≥1.300−≤1.699	≥1.700−≤1.899	≥1.900		
*C. albicans*	52	1	18	10	12	11 (21,2%)	
*C. parapsilosis*	43		17	15	9	2 (4,7%)	
*C. glabrata*	8			5	2		1^a^
*C. tropicalis*	7			5	2		
*C. guillermondii*	6		3	3			
*C. krusei*	1		1				
*C. metapsilosis*	2		1	1			
*C. inconspicua*	1	1					
*C. orthopsilosis*	1						1^b^
*C. neoformans*	1			1			
*M. capitatus*	1		1				
*R. mucilaginosa*	1			1			
	124	2	41	41	25	13	2

*^a^Misidentified as C. krusei with score value 1.200 and two consecutive identical species ID in the list of matches. ^b^Misidentified as C. lusitaniae with score value 1.130 and two consecutive identical species ID in the list of matches. ^∗^Results were compared with those obtained by MALDI-TOF MS from isolated colonies.*

To assess the reproducibility of the rapid procedure, yeast pellets were tested in duplicate by MALDI-TOF MS. The results revealed 100% agreement on species identification, score range and number of consecutive repetitions in the list of matches.

The results of AFST obtained by the rapid procedure were compared with those using the procedure currently used in our laboratory. Essential agreement (EA), i.e., MIC difference of ±one twofold dilution, was observed for all the analyzed strains, including all the unidentified and misidentified strains, with all the tested antifungal drugs.

Minimal inhibitory concentration agreement, i.e., same MIC value, ranged from a minimum of 88.7% (110/124) with itraconazole to a maximum of 95.2% (118/124) with anidulafungin and micafungin and, in the overall, averaged 92.7% (1035/1116) for all the yeast/drug combinations tested (**Table 2**). Importantly, MIC discrepancies did not correspond to differences in clinical category agreement for all antifungal agent/yeast species combinations for which breakpoints are available.

**Table 2:** MIC values of yeast strains tested with the rapid procedure in comparison to those isolated on solid media from positive blood cultures.

Yeasts species (number of strains)	MIC agreement^1^ (%) with antifungal									
	AMB B^2^ (0,12–8)	AND (0,015–8)	MIC (0,008–8)	CAS (0,008–8)	5-FC (0,06–64)	POS (0,008–8)	VOR (0,008–8)	ITRA (0,015–16)	FLU (0,12–256)	All antifungals
All tested yeasts (124)	112/124 (90.3%)	118/124 (95.2%)	118/124 (95.2%)	117/124 (94.4%)	116/124 (93.5%)	114/124 (91.9%)	116/124 (93.5%)	110/124 (88.7%)	114/124 (91.9%)	1035/1116 (92.7%)
*C. albicans* (52)	48/52 (92.3%)	49/52 (94.2%)	49/52 (94.2%)	49/52 (94.2%)	50/52 (96.2%)	49/52 (94.2%)	49/52 (94.2%)	47/52 (90.4%)	49/52 (94.2%)	439/468 (93.8%)
*C. parapsilosis* (43)	38/43 (88.4%)	40/43 (93%)	40/43 (93%)	40/43 (93%)	39/43 (90.7%)	36/43 (83.7%)	38/43 (88.4%)	35/43 (81.4%)	36/43 (83.7%)	342/387 (88.4%)
*C. glabrata* (8)	6/8	8/8	8/8	8/8	6/8	8/8	8/8	7/8	8/8	67/72 (93.1%)
*C. tropicalis* (7)	7/7	7/7	7/7	6/7	7/7	7/7	7/7	7/7	7/7	62/63 (98.4%)
*C. guillermondii* (6)	5/6	6/6	6/6	6/6	6/6	6/6	6/6	6/6	6/6	53/54 (98.1%)
*C. krusei* (1)	1/1	1/1	1/1	1/1	1/1	1/1	1/1	1/1	1/1	9/9
*C. metapsilosis* (2)	2/2	2/2	2/2	2/2	2/2	2/2	2/2	2/2	2/2	18/18 (100%)
*C. inconspicua* (1)	1/1	1/1	1/1	1/1	1/1	1/1	1/1	1/1	1/1	9/9
*C. orthopsilosis* (1)	1/1	1/1	1/1	1/1	1/1	1/1	1/1	1/1	1/1	9/9
*C. neoformans* (1)	1/1	1/1	1/1	1/1	1/1	1/1	1/1	1/1	1/1	9/9
*M. capitatus* (1)	1/1	1/1	1/1	1/1	1/1	1/1	1/1	1/1	1/1	9/9
*R. mucilaginosa* (1)	1/1	1/1	1/1	1/1	1/1	1/1	1/1	1/1	1/1	9/9

*^1^MIC agreement: same MIC value. ^2^AMB B, amphotericin B; AND, anidulafungin; MIC, micafungin; CAS, caspofungin; 5-FC, 5-fluorocytosine; POS, posaconazole; VOR, voriconazole; ITRA, itraconazole; FLU, fluconazole. The numbers in parentheses represent the ranges of concentration of the tested antifungal drugs.*

Only four isolates showed resistance to antifungals. In particular, one *C. parapsilosis* resulted resistant to voriconazole with a MIC value of 1 μg/mL (CLSI breakpoints: ≤0,12 μg/mL susceptible, ≥1 μg/mL resistant), and resistant to fluconazole with a MIC value of 128 μg/mL (CLSI breakpoints: ≤2 μg/mL susceptible, ≥8 μg/mL resistant); two *Candida tropicalis* strains, both showing intermediate susceptibility to voriconazole with a MIC value of 0.25 μg/mL (CLSI breakpoints: ≤0.12 μg/mL susceptible, 0.25–0.5 μg/mL intermediate, ≥1 μg/mL resistant), and one *C. krusei* obviously resistant to fluconazole (MIC 256 μg/mL).

## Discussion

Rapid yeast identification by MALDI-TOF MS is being used as a useful adjunct method by an increasing number of clinical microbiology laboratories (Marklein et al., 2009; Yan et al., 2011; Spanu et al., 2012; Idelevich et al., 2014; Morgenthaler and Kostrzewa, 2015), although obtaining reliable ID results with yeasts is more challenging than with bacteria. Several approaches have been proposed to increase the correct ID rate of yeasts using MALDI-TOF MS, including additional washing steps (Yan et al., 2011), spotting higher sample amounts on the target plate (Idelevich et al., 2014), and adopting lower score cut-offs (Spanu et al., 2012; Idelevich et al., 2014; Morgenthaler and Kostrzewa, 2015) than those indicated by the systems’ manufacturers, in some instances complemented by additional validation criteria specifically conceived to preserve ID accuracy for yeasts recovered from positive BCs (Idelevich et al., 2014; Morgenthaler and Kostrzewa, 2015). In the present study, the rapid procedure combined with score values ≥1.7 and ≥4 consecutive identical species ID in the list of matches yielded a 100% concordance in species ID with the routine MALDI-TOF MS method, allowing to reliably identifying 53.2% of all the tested yeast strains. Several authors have proposed to lower down cut-off values for MALDI-TOF MS identification of microorganisms directly from positive BCs. In this context, we observed that cut-off values could be lowered down to 1.3 together with ≥4 consecutive identical species ID in the list of matches without compromising accuracy. This would allow extending concordant/correct identification to 107 of 124 (86.3%) isolates.

Direct AFST using Sensititre YeastOne Y010 resulted in 100% EA with the same microdilution method routinely used in our laboratory on isolated colonies. Furthermore, MIC agreement was >90% for all the tested antifungal drugs, with the sole exception of itraconazole (88.7%), reaching the highest percentage with anidulafungin and micafungin (95.2%). In general, higher percentages of MIC agreement were observed with echinocandins than with azoles.

The present study was performed by using a CLSI validated method for AFST. As the CLSI standards do not always correspond to those of EUCAST, in particular for some classes of antifungals (azoles and echinocandins), it would be interesting to perform a study to compare the two systems in order to evaluate possible differences in categorical agreements (susceptible/resistant). In this context, a recent study by Arendrup et al. (2017) compared the EUCAST and CLSI microdilution methods on 123 *Candida auris* isolates, and found significant differences in the MIC values for most antifungal agents tested. Therefore, the applicability and performance of the proposed rapid procedure using the EUCAST method, instead of the CLSI method, deserves further investigation.

Noteworthy, this study was carried out in an area with a relatively low incidence of resistance to antifungal agents. An evaluation of the proposed procedure in an area with higher incidence of resistance to antifungals would be useful to assess its possible general applicability in a broader context. In addition, it would be of interest to evaluate the performance of the proposed rapid procedure in a geographic area where BSI caused by *C. auris* has been reported, as well as in clinical settings where BSIs caused by non-*Candida* yeasts and/or molds are more frequent.

In conclusion, the results of the present study show that the same yeast suspension, recovered from monomicrobial BCs, can be used to perform rapid ID and AFST, yielding accurate and reliable AFST results. Noteworthy, AFST results obtained with the rapid procedure showed EA with those by the routine procedure even for all misidentified and not identified yeast strains. With such a rapid procedure, ID by MALDI-TOF MS is available in less than 1 h and AFST, in most cases, in 24 h from BC positivity, thus allowing reporting the results at least 24 h earlier than with the routine procedure. This information is essential to guide clinicians to the appropriate antifungal therapy, thus allowing reducing mortality, morbidity, and health care costs.

## Author Contributions

AV, SB, and EG conceptualized the data. AV, WF, and FC contributed to experiments execution and analysis of results. WF, AL, and EG drafted the manuscript.

## Conflict of Interest Statement

The authors declare that the research was conducted in the absence of any commercial or financial relationships that could be construed as a potential conflict of interest.
